# Statistical Validation for Clinical Measures: Repeatability and Agreement of Kinect™-Based Software

**DOI:** 10.1155/2018/6710595

**Published:** 2018-03-20

**Authors:** Natalia Lopez, Elisa Perez, Emanuel Tello, Alejandro Rodrigo, Max E. Valentinuzzi

**Affiliations:** ^1^Gabinete de Tecnología Médica, Facultad de Ingeniería, Universidad Nacional de San Juan, CONICET, San Juan, Argentina; ^2^Instituto Superior de Investigaciones Biológicas, CONICET, Tucumán, Argentina

## Abstract

**Background:**

The rehabilitation process is a fundamental stage for recovery of people's capabilities. However, the evaluation of the process is performed by physiatrists and medical doctors, mostly based on their observations, that is, a subjective appreciation of the patient's evolution. This paper proposes a tracking platform of the movement made by an individual's upper limb using Kinect sensor(s) to be applied for the patient during the rehabilitation process. The main contribution is the development of quantifying software and the statistical validation of its performance, repeatability, and clinical use in the rehabilitation process.

**Methods:**

The software determines joint angles and upper limb trajectories for the construction of a specific rehabilitation protocol and quantifies the treatment evolution. In turn, the information is presented via a graphical interface that allows the recording, storage, and report of the patient's data. For clinical purposes, the software information is statistically validated with three different methodologies, comparing the measures with a goniometer in terms of agreement and repeatability.

**Results:**

The agreement of joint angles measured with the proposed software and goniometer is evaluated with Bland-Altman plots; all measurements fell well within the limits of agreement, meaning interchangeability of both techniques. Additionally, the results of Bland-Altman analysis of repeatability show 95% confidence. Finally, the physiotherapists' qualitative assessment shows encouraging results for the clinical use.

**Conclusion:**

The main conclusion is that the software is capable of offering a clinical history of the patient and is useful for quantification of the rehabilitation success. The simplicity, low cost, and visualization possibilities enhance the use of the software Kinect for rehabilitation and other applications, and the expert's opinion endorses the choice of our approach for clinical practice. Comparison of the new measurement technique with established goniometric methods determines that the proposed software agrees sufficiently to be used interchangeably.

## 1. Background

World Health Organization (WHO) [1] reported that more than one billion people in the world live with some form of disability; out of this number, nearly 200 million experience considerable difficulties in their daily functions. In the years ahead, disability will be an even greater concern because its prevalence is on the rise. This is due to ageing populations and the higher risk of disability in older people as well as the global increase in chronic health conditions, such as diabetes, cardiovascular disease, cancer, and mental health disorders. The rehabilitation process is a fundamental stage for recovery, helping patients to acquire (or reacquire) new abilities for daily activities and improve independence. Rehabilitation needs an interdisciplinary staff and an exhaustive analysis of the underlying level of impairment and needs of the patient, suited by a thorough planning. However, the evaluation of the process is performed by physiatrists and medical doctors, mostly based on their observations, that is, a subjective appreciation of the patient's evolution. Throughout the last years, several sensing systems have been developed in order to quantify the movements performed by the patient, especially focused on gait analysis, for example, the trademarks called* Codamotion* [2] or* Vicon*™ [3]. These systems have been designed primarily for athletes but they are applicable to disabilities, too. They usually track and analyze 3D movements in every kind of environment, from large fixed laboratories through outdoor sports activities.

Recent studies of the mechanisms underlying plasticity and recovery following neurological injuries have originated innovative lines of research in neurorehabilitation. Additionally, the development of new technologies of performance evaluation and intervention procedures has stimulated research on novel rehabilitation paradigms and more effective strategies. Nonetheless, their transfer into actual clinical practice remains a challenge, meaning that further research to assess their effectiveness is needed [4].

Reduced mobility of upper limbs, whatever its origin, makes the development of a diagnostic evaluation tool for movement quantification imperative. Motion capture has been widely used for rehabilitation exercises, gait analysis, and posture disorders diagnosis. Artificial vision systems [5, 6] are a suitable alternative or complement to electromyographic acquisition [7, 8], inertial measurement devices, goniometry, and other techniques, mainly because of their low cost, noninvasiveness, and user's comfort.

Kinect is a line of motion sensing input devices by Microsoft, originally intended for video games and first introduced in 2010. Interesting is the fact that, besides its entertainment purposes, it can serve as evaluation and physical rehabilitation system. The authors in [9, 10] used Kinect to motivate adolescents that suffer from cerebral palsy to carry out their rehabilitation process. In [11], the authors applied a virtual reality system using cameras for hand and arm rehabilitation. The patient performs the task and the system follows the movements of the hand and arm, generating audible feedback when the patient achieves the goal. The system senses the movement range and velocity but without validation [12]. Other uses of Kinect include sensing systems for patients with lateral disorders and detection and follow-up of right and left hand and face movements. Majority of applications for rehabilitation use visual feedback and gaming incentives based on virtual reality to promote exercise therapy, improving patient's movement abilities. There are many references available, for example, [13], which dealt with the upper limb reachable workspace determined by the use of Kinect or [14] that uses the same sensor to evaluate functional scores in stroke patients with an objective perspective by means of correlation coefficients. As was pointed out by [15], the anatomical landmark of Kinect has excellent validity with the data obtained by a 3D camera-based system. For rehabilitation purposes and motivation for exercise, a Kinect-based system was tested in two young adults in a public school setting, with encouraging results [16]. The work of Rammer et al. [17] presents a study with 12 adolescents that were evaluated using both the Kinect-based system and the Shriners Hospitals for Children Upper Extremity Evaluation (SHUEE), also a subjective measure method. With statistical correlation analysis, they concluded that Kinect sensor can be applied to standardized task-based tests. Similarly, in Vernon et al.'s work [18], the reliability measures obtained with Kinect sensor are examined and compared with the performance on common clinical tests in stroke patients. They assessed the test-retest reliability using intraclass correlation coefficient, redundancy with Spearman's correlation, and score prediction on the clinical tests using multiple regressions. Their results demonstrate that the use of Kinect may provide reliable and clinically useful information.

In [9], a comparison with Vicon motion capture demonstrated the usability of Kinect to record movement and posture, reporting the need of a further development that allows portable ergonomic assessments. Low-cost sensor, plug and play functioning, and open-source code have potentially made of Kinect a useful tool in rehabilitation engineering, but the exhaustive analysis of measurements and comparison with standard methods are necessary before clinical application.

This paper describes a tracking platform of the movement made by an individual's upper limb using Kinect sensor to be applied for the patient during the rehabilitation process. Assessment of the patient's upper limb movement is achieved, extracting a quantification of the movement to provide the physiotherapist or attending physician with more precise data to carry out an objective diagnosis and, thus, plan a more accurate rehabilitation in accordance with each patient's needs. In addition, the evolution can be assessed after the patient begins with the rehabilitation and verify whether the patient is performing the best proposed trajectory, redesigning the therapy if necessary. Only trunk posture and upper limb are included in this paper, which have acceptable performance [19].

The main contribution of the present work is the development of quantifying software and the statistical validation of its performance, repeatability, and clinical use in the rehabilitation process. Therefore, this paper aimed at verifying the usefulness and agreement of measurements obtained through ad hoc software and a goniometer.

## 2. Methods

### 2.1. Kinect Architecture

The Kinect sensor allows the user to control and interact with the console without a traditional video game controller; it uses, instead, a natural interface that recognizes gestures, voice commands, objects, and images. The device has a RGB camera (CMOS technology), a depth sensor, multiarray microphone, and an exclusive processor, all of which provide a 3D movement capture of the whole body as well as facial and voice recognition commands. In this work, we use the first version of the Kinect sensor, developed for Xbox 360 console.

The system has an image processor to generate and keep in memory the image frame of depth and color at a velocity of 30 frames per second (fps). The depth camera acts as a 3D scanner to carry out a tridimensional reconstruction of the scene. This device is composed of an infrared (IR) camera and a structured light infrared projector. A pattern of infrared points is scattered projecting into the scene, over individuals and objects, while the image of such projection is obtained through the infrared light.

The distortion of the IR pattern allows the tridimensional reconstruction of the scene and the depth image, thus creating a grayscale map of 640 × 480 pixels. The whiter colors indicate that the object is further away, while the darker ones indicate that the object is closer [20].

The Windows SDK™ software for Kinect (a set of software tools for developers) allows up to a maximum of 20 joints to define a standing skeleton. It can also delimit or mark off a sitting skeleton providing a maximum of 10 joints. This application works on dynamic situations of the trunk and upper limb, thus establishing the position of the head, breastbone, shoulders, elbows, and right and left wrists throughout the movement.

**Figure 1:**
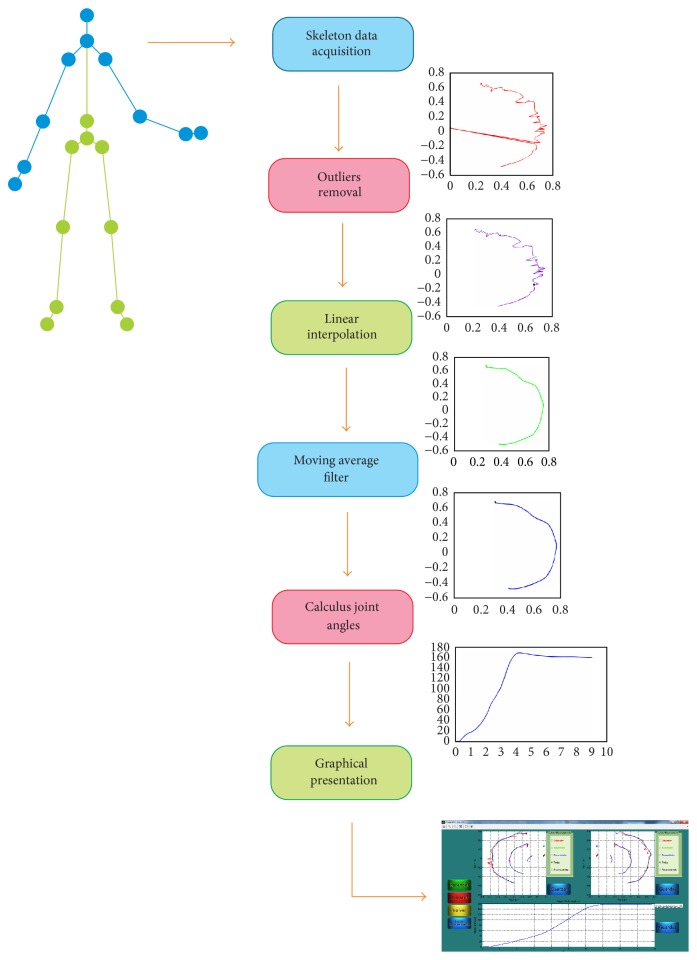
Processing stages, represented by a typical curve that shows the modified acquired data for the purposes of analysis. The software involves all stages and can be accessed through the graphical interface. Raw and processed data can be accessed through the visualization menu.

The software developed implied data processing of the information provided by Kinect. In the first stage, atypical values were eliminated (outliers). In the second stage, the trajectory was processed through the application of linear interpolation algorithms and moving average filters. During the last stage, joint position angles were calculated. These stages will be explained in the next subsections and are briefly described in the block diagram of Figure 1.

#### 2.1.1. Removal of Atypical Values

Points with very different values of the mean value produce large errors during data recording and can lead to unwanted effects on the representation. These effects are mainly seen when the device has to register overlapped points, especially over the longitudinal axis of the camera depth. In this case, Kinect cannot accurately detect the joint, and only an approximate value is inferred, depending on the movement being performed. These atypical values (or* outliers*) are observations that deviate from the rest in such a way that they might have been generated by a different mechanism and indicate experimental error.

To detect and correct outliers, let us assume the measurements as a random sample following a Gaussian distribution quite closely, since 5% of the values in a population are more than 1.96 standard deviations (*σ*) from the mean $\overset{-}{x}$. Under this assumption, this limit can be used to detect the outlier, analyzing the data in consecutive segments of three elements and the calculus of threshold values *L*1 and *L*2. They are defined by (1) and (2); that is,

(1) L1=x−−1.96σ,

(2) L2=x−+1.96σ,

where $\overset{-}{x}$ is the mean calculated between the data centered on the segment and its two adjacent neighbors (*x*_*i*−1_, *x*_*i*_, *x*_*i*+1_), while *σ*  stands for the standard deviation. When the outlier value is lower than the lower threshold *L*1 (see (1)) or higher than the upper threshold *L*2 (see (2)), it is replaced by its neighbors' mean. This procedure was carried out with the data obtained from the three dimensions.

(3) $$\begin{array}{c} x_{i}\left\{\begin{array}{cc} \overset{-}{x}, & \text{if }x_{i}<L1,\\ \overset{-}{x}, & \text{if }x_{i}>L2,\\ x_{i}, & else. \end{array}\right. \end{array}$$

#### 2.1.2. Linear Interpolation

As part of the processing stage, the data underwent a linear interpolation process that can be understood as a weighted average. Each trajectory was organized in groups of 10 values. The interpolation was carried out between the first and last values. The 8 calculated points were replaced by the 8 values of the original trajectory to keep the original data point number. The selection process of the points considered for the interpolation did not widely modify the morphology of the original trajectory.

#### 2.1.3. Filtering Stage

After the interpolation process, a moving average filter was applied, therefore allowing the creation of averages from several subgroups within the complete data set. Each number from the whole group of results represents the average of certain subgroup corresponding to the total data; that is,

(4) $$\begin{array}{c} \overset{-}{x}=\frac{\sum_{i=1}^{n}x_{i}}{n}, \end{array}$$

where $\overset{-}{x}$ is the average value of the subgroup of values *x*_*i*_, formed by *n* samples. In this way, noise was better filtered out, without modifying the real movement trajectory. It was essential that the filter did not alter the movement trajectory but it had to improve its graphic visualization and interpretation.

#### 2.1.4. Angle Calculation

Joint angles were calculated using vectors, depending on the position of each joint, considering that the reference system is located in the Kinect sensor and the three anatomical planes: sagittal, coronal, and axial. Since the movements are acquired frame by frame, capture and representation of joint trajectory were displayed under the same criteria.

**Figure 2:**
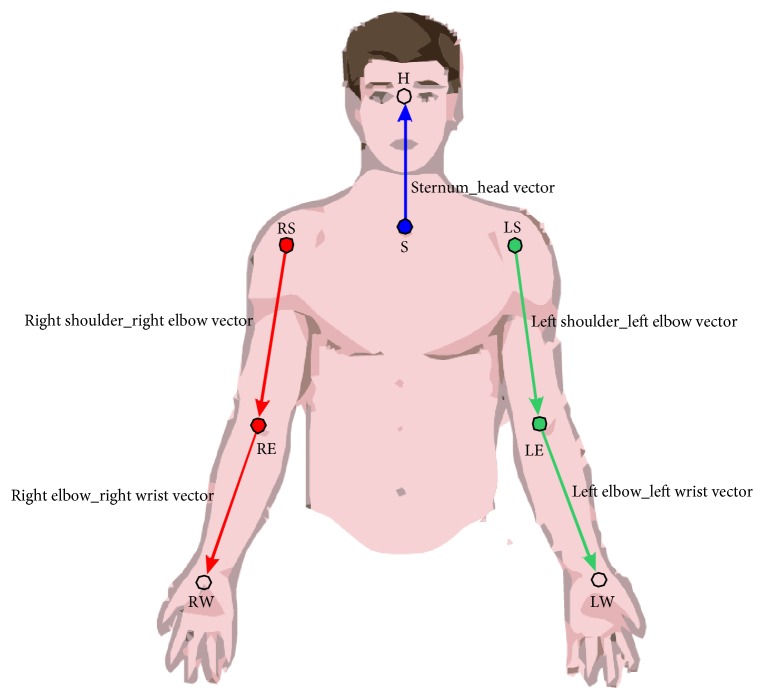
Vector representations to calculate the angles for different joints.

First, we have to define the body segments *P*_*i*_ corresponding to trunk, shoulders, arm, forearm, and hand, as Figure 2 shows.

Calculation was carried out through the classical definition of angle between two vectors; that is,

(5) $$\begin{array}{c} \Theta =\cos^{-1}\frac{P1\cdot P2}{\left\|P1\right\|\cdot \left\|P2\right\|}, \end{array}$$

where *P*1 · *P*2 is the inner product between *P*1 and *P*2 and ‖*P*1‖ and ‖*P*2‖ are their respective norms.

The subscript indicates two consecutive segments, while *θ*, the angle, can be obtained for each movement. Thereafter, the processed data were used to compute the angular position of each joint and for each arm. Besides, the joints excursion for a specific movement was plotted.

### 2.2. Development of the Graphic Interface

A graphic interface (DEMOVA™, i.e.,* Determinación de Movimiento y Valoración Angular, in Spanish, or Movement Determination and Angular Assessment*) was developed to facilitate data management, acquisition, and collection by the medical team. This process enabled easy access to information as well as storage of patients' medical, technical, and personal information. This tool extends the use of the sensor outside the scope of research and brings it closer to clinical practice. Matlab 7.0 was the software employed.

#### 2.2.1. Window Configuration

This display established the initial parameters to carry out the acquisition, mainly the person's right location and the camera tilt, in order to capture its movement more efficiently. This window makes the information of both the RGB and depth cameras available (both of which can be turned on and off indistinctly). Besides, it is the starting point of the remaining interfaces, since, through this calibration process, it is possible to have access to the complete programmed movements.

#### 2.2.2. Acquisition Window

**Figure 3:**
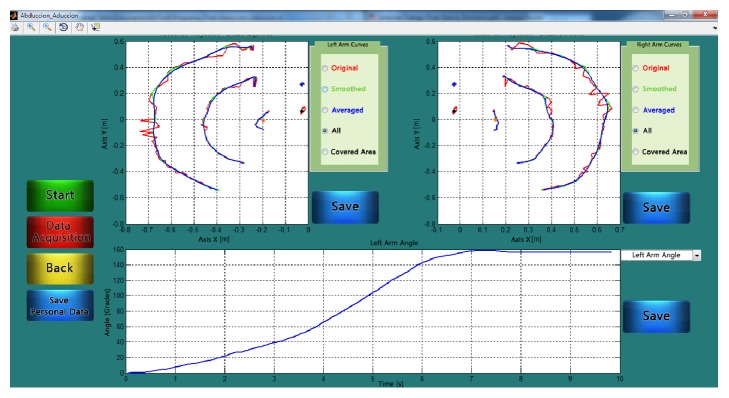
Graphic interface for the DEMOVA software: acquisition window in an abduction-adduction movement. The raw, smoothed, and averaged curves are presented in different colors and the user can choose the presentation through the submenu on the right. The bottom window displays the angle excursion for all joints, selected in the dropdown submenu.

The windows that enabled the trajectory acquisition are separated under the type of movements carried out and in accordance with the required data. In general terms, all the windows have these common sections: graphs on which the trajectory are displayed; buttons: “Start,” “Data Acquisition,” “Back,” “Save Personal Data,” and “Save” (which can also save graphs if required); selection panels to choose options to see the graphs: “Original Curves,” “Smoothed Curves,” “Averaged Curves,” “All the Curves,” and “Covered Area” (Figure 3). Moreover, a second window shows trajectories in the 3D space and the surface covered for the movement.

### 2.3. Experimental Protocol

The complete validation was performed in three stages. For experimental* Protocols 1* and* 2*, nine adults (29.2 ± 3.8 years old, 1.69 ± 0.11 meters high) participated (5 females and 4 males), without history of neurological and/or muscular disorders. The experiments were performed according to the ethical considerations and after signing the informed consent.

**Figure 4:**
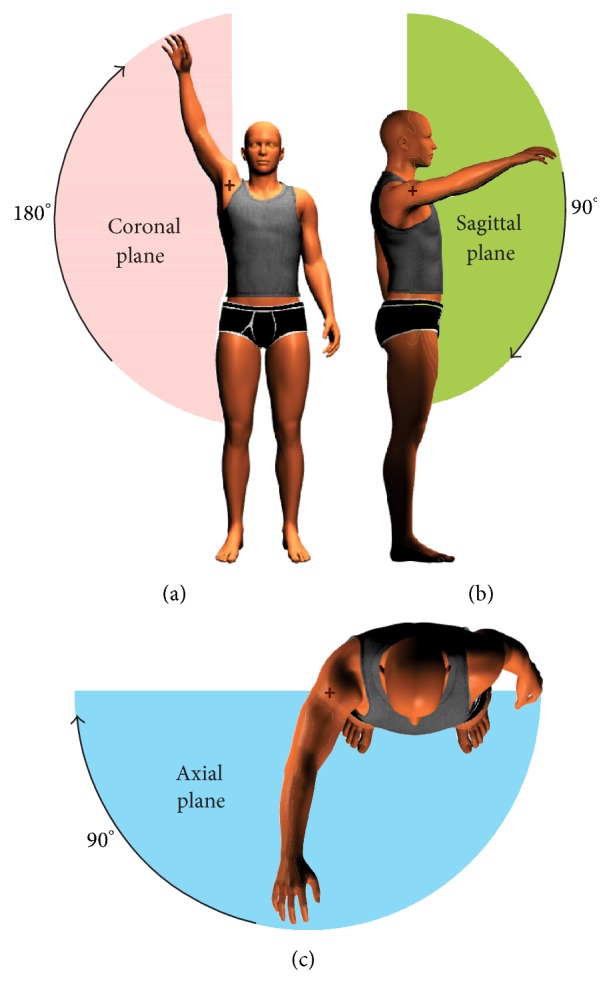
Movements corresponding to Protocol 1 (a) and Protocol 2 (b and c). The abduction movement is performed in the coronal plane and the axis is marked with a cross. Similarly, the figure shows the flexion-extension in sagittal and axial plane for Protocol 2.

The first stage* (Protocol 1)* was a comparative routine for static measurements of the upper limb, carried out by an expert physiotherapist using a goniometer and the DEMOVA software. Its objective was the statistical determination of agreement between the system proposed and the standard method. The volunteers were instructed to assume static positions every 10 degrees with both arms in an abduction-adduction trajectory. The angular position of the shoulder was measured with a goniometer and with the DEMOVA software. The movement was run in the coronal (*x*-*y* projection) because it is the worst condition in terms of Kinect precision, as can be seen in Figure 4(a). The laser depth sensor has a very good performance with intrinsic characteristics (reported error: 14.1–34.8 mm, [17]), so the evaluation was focused on the aforementioned projection.

*Protocol 2* was designed with the aim of evaluating DEMOVA's repeatability. The acquisition results were assessed, repeating the same movement twice. Consequently, a specific movement protocol was followed to develop acquisition and determination of the trajectory, according to the clinical evaluation usually performed by a physiotherapist. The volunteers were asked to carry out the suggested tasks at a comfortable speed. The individuals were placed in front of the camera (2 meters, recommended by Kinect) and completed the set of movements. The protocol included shoulder flexion in both planes, sagittal and axial at 90 degrees (Figures 4(b) and 4(c)). All movements were performed in two different sessions by each user to examine their repeatability.

Finally, in* Protocol 3*, a qualitative assessment of the software was performed through a questionnaire answered by three physiotherapists in order to evaluate the data presentation, graphical interface, and overall performance of DEMOVA as a clinical tool (Table 1).

### 2.4. Statistical Analysis

The most commonly used technique for measuring joint angles and upper limb motion is the goniometer, long considered the gold standard. It is important to note that both measures (by Kinect and by the goniometer) are estimates of the real one because they are indirect and noninvasive techniques. Besides, the goniometer measure is affected by the observer subjectivity and can vary in different sessions.

To test validity, we evaluated the agreement between DEMOVA and the goniometric measurements using Bland-Altman plots [21]. Two measurements of the same subject performed with different methods may differ for several reasons but, when trying to focus on the method itself (and not on population),* agreement* is the characteristic that better describes how close the two measurements are. As pointed out by Bland and Altman,* agreement* is the correct approach when the true value is unknown. Significance and correlation are not adequate. Under the Gaussian hypothesis, it can be assumed that 95% of the data are within the range that lies between the limits of agreement; that is,

(6) inf=m−−1,96×sdd,sup=m−+1,96×sdd,

where *m* is the mean of both measures (goniometer and DEMOVA) and *d* is the difference between them. If this hypothesis is met, it is valid to affirm that the two methods are interchangeable. The Bland-Altman plot was constructed by using the data of the first experimental protocol (see below).

Repeatability is defined as the closeness between the results of the same measurement under repeatable conditions, with the same procedure, by the same observer and the same instrument. Dispersion is a good way for such assessment. For that matter, Protocol 2 was applied and the mean and standard deviations of the data obtained with DEMOVA in two sessions were analyzed. Once again, assuming a Gaussian distribution, (6) was modified replacing *m* by *u*, the difference between two measurements of the same subject in two sessions; that is,

(7) infRep=u−−1,96×sdu,supRep=u−+1,96×sdu.

These limits are according to the repeatability concept adopted by the British Standards Institution [22], in which 95% of differences are expected to be less than 2*sd*. This coefficient CR can be easily computed, assuming the mean difference to be zero (or near to zero):

(8) $$\begin{array}{c} CR=\sqrt{\frac{\sum \left(u^{2}\right)}{n}}. \end{array}$$

## 3. Results

### 3.1. Agreement

**Figure 5:**
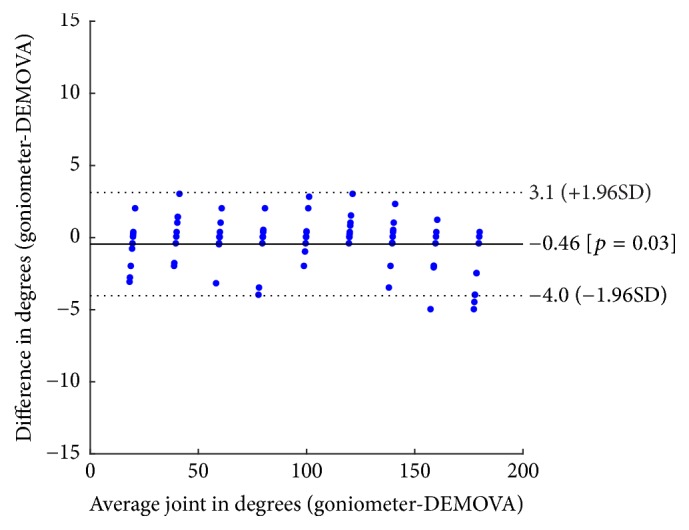
Bland-Altman plot of mean difference for the shoulder joint (0 to 180°) against its mean (experimental Protocol 1). Mean difference indicated by solid line (−0.46) and the 95% limits of agreement shown by dashed lines. No outliers outside the limits.

The agreement of joint angles measured with DEMOVA and goniometer was evaluated with Bland-Altman plots (Figure 5). The mean difference between DEMOVA and the goniometric measurements was −0.46°, while the limits given by the confidence interval (see (6)) were 3.1° and −4.0°. Such limits are consistent with the instrumental error reported for Kinect [17]. The absence of outliers is remarkable; in other words, all measurements fell well within the limits of agreement, indicating interchangeability of both techniques. Therefore, 95% of future differences would lie within these limits.

### 3.2. Repeatability

**Figure 6:**
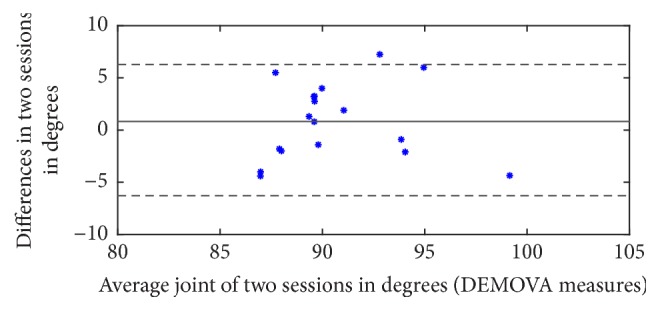
Bland-Altman analysis of repeatability. Mean value = 0.83 and bounds of confidence interval (dotted lines) ±6.28. There is a single outlier in these measurements.

Figure 6 shows the results of Bland-Altman analysis of repeatability, with 95% confidence interval (±6.28). These results belong to the experiments performed with Protocol 2, measurements with DEMOVA in two sessions, for all volunteers in 90 degrees position.

With these results, we can infer that, in next sessions using DEMOVA, 95% of the obtained values will be in the confidence interval, in this case between 84° and 96° degrees (approximately). This conclusion complies with agreement results, because all the analysis is limited by Kinect intrinsic errors.

Variability in measurements made on the same subject can be ascribed only to errors in the measurement method, so the couple of measures that we can see out of the boundaries of the confidence interval can be attributed to changes in lighting conditions and sensor errors rather than angle calculus. The coefficient of repeatability is CR = 3.6406, calculated with (8), which means that, in future measurements made by DEMOVA method under identical conditions, the results will have variations less than 4.04% in any range of measure.

### 3.3. DEMOVA Qualitative Assessment

After completing the questionnaire, the physiotherapists' qualitative assessment was scored by 40-42-29; that is, two of them evaluated DEMOVA as a trustworthy tool and the third one considered it as a useful tool but it does not inspire enough confidence to replace the goniometer method completely. The questionnaire model is shown in Table 1.

Regarding the information provided by the DEMOVA plots, all participants point out the easy understanding of asymmetries, joint angles and restrictions, trunk compensations, and time evolution information. This kind of data is unavailable in conventional measures and can be stored for further analysis of the patient.

From the transcripts, the authors identified the novelty as the most important motivator to the use of DEMOVA. Patient's safety, clarity of visualization, research possibilities, and objective analysis of the patient's evolution were central to these data. Interviewees also made open suggestions for future improvements of the software.

## 4. Discussion

In this paper, we propose software based on the new Kinect technology to acquire and quantify movements, as well as the determination of upper limb joint angles, which are offered to the specialist to improve the diagnosis during the global rehabilitation process. The software is capable of offering a clinical history of the patient useful for quantification of the rehabilitation success.

The data were processed to produce a smoothed trajectory, removing noise and outliers values but preserving the natural behavior of each user. To present the patients' data, a graphical interface was used, which included algorithms that enhance the visualization of the movements performed throughout the different processing stages as well as the angles' graphics. It is worth mentioning that the software does not require sophisticated equipment, thus facilitating its use in any personal computer. The system is of low cost and is easy to use, two characteristics that make it suitable for everyday use by physical therapists and physicians. In addition, the movements can be personalized to the patient's needs by adapting the system to the range of motions and tasks.

Comparison of the new measurement technique with established goniometric methods is often needed to determine whether the DEMOVA software agrees sufficiently to be used interchangeably. In this order, the statistical analysis with Bland-Altman plots is more appropriate than correlation coefficient or regression [20], especially with indirect or estimated measures, in which the agreement between both measurement techniques is a must. As presented in Figures 4 and 5, all measures were between the limits of agreement, allowing the use of DEMOVA software instead of the goniometer. It is important to note that measurements with goniometer vary with observer expertise, scapular motion, and identification of anatomical structures or the reference points. Kinect and the designed software have intrinsic errors due to image capture and uncertainties, which can be minimized with correct positioning, fixed distance to the sensor, and clarity in verbal commands. However, simplicity, low cost, and visualization possibilities enhance the use of DEMOVA and Kinect for rehabilitation and other applications, and the expert's opinion endorses the choice of our approach for clinical practice.

## Figures and Tables

**Table 1:** Total scores: 0–10: the software is evaluated as useless; 10–20: DEMOVA cannot replace goniometer measures; 20–30: DEMOVA is considered as a complementary tool for diagnosis and evaluation of a patient, alternative to goniometer; 30–45: DEMOVA is considered as a reliable tool for quantitative analysis of movement.

Related to graphic interface of DEMOVA					

(1) The software was simple to use?	Select one option: 1 = very difficult; 2 = difficult; 3 = normal; 4 = easy; 5 = very easy				
	1	2	3	4	5

(2) Would you usually use it as a measurement tool?	Select one option: 1 = never; 2 = sometimes; 3 = oftentimes; 4 = usually; 5 = always				
	1	2	3	4	5

(3) The information is clearly distributed in the screen?	Select one option: 1 = no; 2 = poor; 3 = normal; 4 = good; 5 = very good				
	1	2	3	4	5

(4) Do you think that the soft is user friendly?	Select one option: 1 = no; 2 = a little; 3 = normal; 4 = friendly; 5 = very friendly				
	1	2	3	4	5

Related to information					

(5) Analysis and display of movement paths of upper limb in the three planes is easy to understand?	Select one option: 1 = no; 2 = difficult; 3 = normal; 4 = simple; 5 = very simple				
	1	2	3	4	5

(6) Do you think that trajectories graphic information is a useful tool to complete your diagnosis and clinical evaluation?	Select one option: 1 = no; 2 = a little; 3 = normal; 4 = useful; 5 = very useful				
	1	2	3	4	5

(7) Do you think that you can replace goniometric measures with DEMOVA in the clinical evaluation?	Select one option: 1 = no; 2 = maybe; 3 = I will use both; 4 = I will use DEMOVA and in occasions the goniometer; 5 = replacement for DEMOVA				
	1	2	3	4	5

(8) Do you consider the 3D graphic of (area) surface covered for upper limb useful for analysis purposes?	Select one option: 1 = no; 2 = a little; 3 = normal; 4 = useful; 5 = very useful				
	1	2	3	4	5

(9) Graphics of abduction-adductions of two patients are presented in this option. One of them has upper limb disturbances. Can you describe the information as relevant in the comparison? 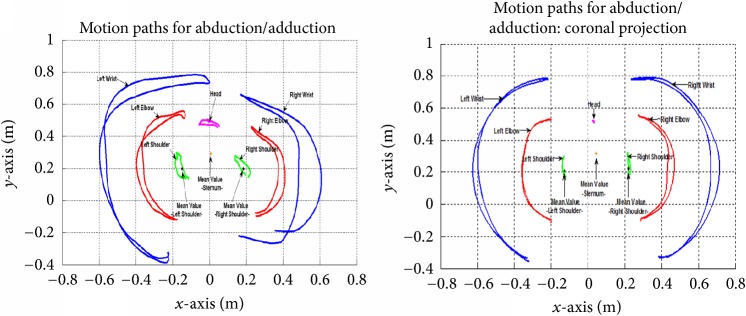	Please, explain your opinion				

(10) Related to item (9): is the graphical information relevant for quantitative evaluation of the affected patient?	Select one option: 1 = no; 2 = a little; 3 = normal; 4 = useful; 5 = very useful				
	1	2	3	4	5

(11) Do you think that DEMOVA is a useful tool for diagnosis, quantitative evaluation, and long-term treatment for upper limb movements?	Please, explain your opinion				
